# Determination of chitinase 3-like 1 in cerebrospinal fluid in multiple sclerosis and other neurological diseases

**DOI:** 10.1371/journal.pone.0233519

**Published:** 2020-05-21

**Authors:** Pavlína Kušnierová, David Zeman, Pavel Hradílek, Olga Zapletalová, David Stejskal

**Affiliations:** 1 Department of Clinical Biochemistry, Institute of Laboratory Diagnostics, University Hospital Ostrava, Ostrava, Czech Republic; 2 Clinic of Neurology, University Hospital Ostrava, Ostrava, Czech Republic; University of Rome Tor Vergata, ITALY

## Abstract

**Objectives:**

Chitinase 3-like 1 (CHI3L1) is an extracellular monomeric single-chain glycoprotein expressed by many types of cells. Its elevated levels were found in cerebrospinal fluid in central nervous system (CNS) inflammatory diseases patients. The aim of the study was 1) to validate the reference interval of cerebrospinal fluid (CSF) CHI3L1 in a control group; 2) to measure the CHI3L1 concentration in different diagnosis groups .including multiple sclerosis (MS); and 3) to correlate those values with other biomarkers of axonal damage or neuroinflammation in different grous.

**Methods:**

The study included 132 CSF samples sent to the Department of Clinical Biochemistry, Institute of Laboratory Diagnostics, University Hospital Ostrava. Concentrations of CHI3L1, CXCL13 chemokine, neurofilament light chains, and phosphorylated neurofilament heavy chains were determined by enzyme-linked immunosorbent assays. IgG oligoclonal bands were detected by isoelectric focusing in agarose gels followed by immunofixation. IgM and FLC oligoclonal bands were analyzed by IEF followed by affinity immunoblotting. The group consisted of 42 patients with multiple sclerosis, 14 with clinically isolated syndrome, 11 with other central nervous system inflammatory diseases, 46 with non-inflammatory diseases of the central nervous system, 4 with inflammatory diseases of the peripheral nervous system, and 15 controls.

**Results:**

The estimated reference values of CHI3L1 were 28.6–182.5 μg.L^-1^. Statistically significant differences of CSF CHI3L1 concentrations were found among diagnosis groups (p < 0.0001), after age adjustment (p = 0.002). There was a statistically significant relationship between CHI3L1 and NFL in the MS group (r_s_ = 0.460; P = 0.002), and between CHI3L1 and pNFH in the MS group (r_s_ = 0.691; P < 0.001). No statistically significant difference was found in the categorical comparison of CHI3L1 in the MS group and other diagnostic groups as well as when using the Mann-Whitney U test for CHI3L1 with additional parameters with and without oligoclonal bands present.

**Conclusions:**

CSF CHI3L1 values differ depending on diagnosis and correlate significantly with concentrations of the axonal damage markers CSF neurofilament light chains, and CSF phosphorylated neurofilament heavy chains, but not with CSF concentrations of the inflammatory marker CXCL13. Thus, CSF CHI3L1 could be another promising prognostic, albeit probably etiologically nonspecific, biomarker of MS.

## Introduction

Multiple sclerosis (MS) is a chronic disease affecting the central nervous system. In recent years several biochemical markers in cerebrospinal fluid have been suggested as prognostic tools [1–3].

CHI3L1, also known as YKL-40, belongs to the chitin glycoside hydrolase 18 family. Unlike true chitinases, it lacks enzymatic activity. It is a glycoprotein produced by a wide variety of cells, such as macrophages, chondrocytes, synovial cells, osteoblasts, neutrophils, and astrocytes [4–6].

CHI3L1 is expressed in astrocytes in the brain tissue of patients with multiple sclerosis, and is associated with reactive gliosis in different neuropathological states, particularly those associated with neuroinflammation. A correlation between the time course of the CHI3L1 concentration and the CSF viral load was shown in lentiviral encephalitis [7]. CHI3L1 is released in vitro from macrophages but the CHI3L1 protein is present in vivo around the microglial nodes in certain astrocytes. CHI3L1 mRNA is expressed by reactive astrocytes surrounding the microglial nodes, suggesting that macrophages release inflammatory mediators that can induce CHI3L1 expression in surrounding astrocytes but not in neurons. The transcription of CHI3L1 by macrophages is likely to be inhibited only after they enter the brain, which may be the cause of the differences observed in other tissue pathologies [8–9].

MS is a demyelinating disease associated with increasing and decreasing inflammation, gliosis, and variable axonal loss. Therefore, we expect to find increased concentrations of CHI3L1 in MS patients.

The aim of the study was 1) to validate the reference interval (RI) of cerebrospinal fluid (CSF) chitinase 3-like 1 (CHI3L1) in a control group; 2) to measure the CHI3L1 concentration in different diagnosis groups, including MS; and 3) to correlate those values with other biomarkers of axonal damage or neuroinflammation in different groups.

RIs were estimated on the basis of the guidelines of the Clinical and Laboratory Standards Institute (CLSI C28-A3), which recommends the use of nonparametric tests for statistical data processing and the evaluation of data according to gender and age [10–11].

## Materials and methods

### Patients

Our study includes 132 patients of the Moravian-Silesian region of the Czech Republic whose CSF samples were sent for analysis to the Institute of Laboratory Diagnostics, Department of Clinical Biochemistry, University Hospital Ostrava. Informed consent was obtained from all patients at the University Hospital Ostrava who were included in the study. The study was approved by the Ethics Committee of the University Hospital Ostrava as a part of the project ‘CSF biomarkers of multiple sclerosis’ (reference number 400/2017). Patients were subdivided into diagnosis groups: MS (n = 42; 33 women, average age 39.5 ± 12.1 years; 9 men, average age 36.6 ± 10.5 years), clinically isolated syndrome (CIS; n = 14; 8 women, average age 28.8 ± 8.3 years; 6 men, average age 28.3 ± 8.9 years), other central nervous system inflammatory diseases (OIND; n = 11; 5 women, average age 78.4 ± 21.2 years; 6 men, average age 62.3 ± 12.1 years), inflammatory diseases of the peripheral nervous system (IDPNS; n = 4;; 4 men, average age 53.5 ± 13.3 years), non-inflammatory diseases of the central nervous system (NIND; n = 46; 32 women, average age 51.8 ± 17.1 years; 14 men, average age 59.1 ± 11.1 years), and controls (n = 15; 11 women, average age 41.7 ± 15.2 years; 4 men, average age 40.2 ± 15.5 years). For diagnosis of multiple sclerosis, we used the 2017 Revisions of the McDonald Criteria [12]. 26 patients were diagnosed as relapsing-remitting MS (RR-MS) while 14 patients were classified as primary progressive (PP-MS); in the remaining two patients, the MS course could not be evaluated because no follow-up documentation was available. None of the patients from MS and CIS groups was on disease-modifying drug (DMD) treatment at the time of sample collection. Lumbar puncture was performed within 3 months after the first symptom of an attack in 7 CIS patients and 14 RR-MS patients. The diagnoses in the OIND group comprised neuromyelitis optica (n = 2); encephalitis (n = 1), granulomatosis with polyangiitis (n = 2), aseptic meningitis (n = 2), neuroborreliosis (n = 3), and myelitis (n = 1). The IDPNS group included patients with acute (n = 2) and chronic (n = 2) inflammatory demyelinating polyneuropathy. The NIND group included a very wide and heterogeneous spectrum of diagnoses: the more frequent were neurodegenerative diseases (n = 11), non-inflammatory polyneuropathy (n = 5), vascular CNS disease (n = 19) and vertigo (n = 5), with fewer cases of CNS tumors (n = 3), radiculopathy (n = 1), anisocoria (n = 1), motor neuron diseases (n = 1), spondylogenic cervical myelopathy (n = 2), cervicobrachial syndrome (n = 1), epilepsy (n = 4), tremor (n = 1), and amyotrophy (n = 2). In estimating the reference interval we used 43 controls (the original group of controls (see above, n = 15) was extended by another 28 CSF samples from CSF biobank of the University Hospital Ostrava). The average age was 40.9 ± 15.1 years. There were 30 women, of average age 41.1 ± 13.0 years, and 13 men, of average age 40.5 ± 19.6 years.

### Samples

CSF samples were collected into polypropylene tubes (Sarstedt, Nümbrecht, Germany). Samples were centrifuged at 390 × g for 10 minutes at room temperature, and the supernatants for determination of CHI3L1, CXCL13, neurofilament light chains (NFL), and phosphorylated neurofilament heavy chains (pNFH) were then aliquoted into at least 3 vials (0.3 ml per vial) and stored at -70 °C until analysed.

### Analytical methods

The concentrations of CHI3L1 (Quantikine ELISA Human Chitinase-3-like 1 Immunoassay, REF DC3L10, R&D Systems, USA&Canada), CXCL13 (CXCL13 ELISA, REF EQ6811-9601-L, Euroimmun AG), NFL (NF-light ELISA, REF 10–7001, IVD CE, UmanDiagnostics AB, Umeå, Sweden), and pNFH (Neurofilament “pNf-H” ELISA, REF EQ 6561–9601, IVD CE, Euroimmun AG, Lübeck, Germany) were determined by ELISA. Undiluted CSF was used for CXCL13 and pNf-H whereas 1/2 and 1/50 dilution was used for NFL and CHI3L1, respectively.

The quality control samples supplied by the manufacturers of the diagnostic kits were used for accurate and reproducible measurement of CHI3L1, CXCL13, and pNHF; for NFL measurements, the patient sample was used, because the diagnostic kit did not include a quality control sample. Kit manufacturers reported that analytical sensitivity was 3.55 ng.L^-1^ for CHI3L1, 10.7 ng.L^-1^ for CXCL13, 32 ng.L^-1^ for NFL, and 27 ng.L^-1^ for pNFH. All samples were measured in duplicate. The mean coefficients of variation for CHI3L1, CXCL13, NFL, and pNFH were 5.8%, 5.2%, 1.9%, and 3.3%, respectively.

IgG oligoclonal bands (oIgG) were detected by isoelectric focusing in agarose gels followed by immunofixation, using a commercial kit on a Hydrasys instrument (Hydragel 9 CSF isofocusing, Cat. No. 4355, Sebia).

IgM and FLC oligoclonal bands (oIgM, oFLC) were analyzed by IEF focusing followed by affinity immunoblotting as originally described by Sindic and Laterre [13] and slightly modified by us [14]. Two extra oligoclonal bands (OCB) in CSF compared to serum (OCB ≥ 2) were the interpretation criteria for intrathecal oligoclonal IgG, IgM, and FLC synthesis [15].

### Statistical methods

The statistical analysis was rendered using Excel, Stata version 13, MedCal version 17.9.7., R and NCSS 2007 software [16–17]. MedCal version 17.9.7.was applied to estimate the CHI3L1 reference interval too. The robust CLSI C28—A3 method was used, due to the small sample size (n = 43).

Basic descriptive statistics, including frequency tables, medians, arithmetic means, standard deviations and percentiles, were used to describe the results. With the Shapiro-Wilk test of normality, the normality of the parameters CHI3L1, CXCL13, NFL, and pNFH was verified.

The normality hypothesis was rejected; therefore, non-parametric tests were used, including the Kruskal-Wallis rank test and the two-sample Wilcoxon rank-sum (Mann-Whitney) test.

The relationship between the parameters was assessed by Spearman’s correlation coefficient. Data values were classified as positive and negative. Fisher’s exact test was used to test categorized data. Conformity between assay results was measured using the kappa index with confidence intervals of 95%. Statistical tests were evaluated using a significance level of 5%.

## Results

First, we validated the diagnostic kit for CHI3L1 determination. The coefficients of variation were comparable to the values supplied by the manufacturer (Table 1). Based on repeated measurements (n = 6) of the blank and the low-concentration CHI3L1 sample, the limit of blank (LoB = 1.05 ng.L^-1^) and the limit of detection (LoD = 5.48 ng.L^-1^) values were calculated. The average recovery of CHI3L1 obtained using spiked samples of CSF was 101.4% (Table 2).

**Table 1:** Assessment of the precision of commercial CHI3L1 ELISA using the manufacturer’s controls.

Control	Intra-Assay Precision			Inter-Assay Precision		
	Level 1	Level 2	Level 3	Level 1	Level 2	Level 3
n	10	10	10	5	5	5
Mean (ng.L^-1^)	335	1030	2084	417	1109	2094
SD (ng.L^-1^)	15.9	52.2	105.1	22.2	63.0	135.5
CV (%)	4.8	5.1	5.0	5.3	5.7	6.5
CV_d_^†^ (%)	4.7	4.3	4.7	5.3	5.8	6.9

n, number of measurements; SD, Standard deviation; CV (%), coefficient of variation;

^†^the declared value of coefficient of variation from the manufacturer

**Table 2:** The recovery of CHI3L1 obtained from spiked samples of CSF.

Theoretical concentration (μg.L^-1^)	Measured concentration (μg.L^-1^)		Average (μg.L^-1^)	Recovery (%)
	1. measurement	2. measurement		
32.3	34.0	28.3	31.2	96.4
149.8	162.0	158.0	160.0	106.8
267.5	289.0	295.0	292.0	109.2
385.3	340.5	356.0	348.3	90.4
503.0	512.0	535.0	523.5	104.1

**Fig 1:**
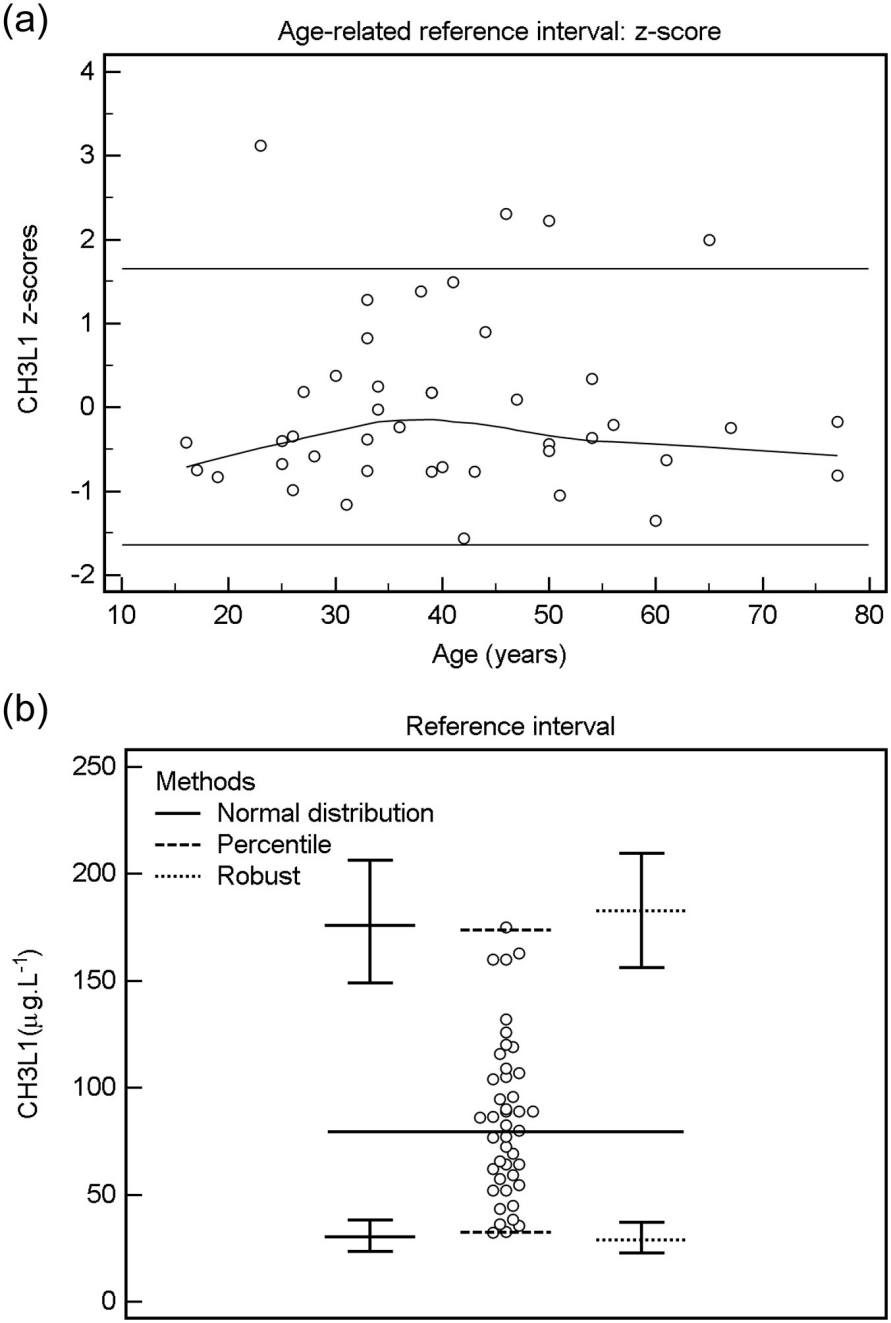
Estimation of the CHI3L1 CSF reference interval and age-dependence of test values. **A:** Age-related reference interval: centiles; **B:** reference interval.

The estimated reference interval (28.6–182.5 μg.L^-1^) showed age-related increase (CHI3L1 = 41.546 + 1,072 * Age, P = 0.0017), sex-related difference was not found (P = 0.837) (Fig 1).

A total of 132 patient samples were included in the analysis, which evaluated the correlation between CHI3L1 levels and several other biochemical markers. Characteristics of the studied groups are presented in Table 3; for summary characteristics please see Supplementary material (S1 Table). Age distribution in diagnostic groups compared with One-way analysis of variance (ANOVA) was statistically different (p < 0.05).

**Table 3:** Descriptive characteristics of the studied groups.

MS						
Variable	n	Min	Max	Mean	Median	SD
Age (year)	42	18.00	69.00	38.833	38.00	11.95
CSF CHI3L1 (μg.L^-1^)	42	35.70	392.00	139.35	130.50	68.80
CSF NFL (ng.L^-1^)	42	97.00	4044.00	1036.45	721.00	875.43
CSF pNFH (ng.L^-1^)	35	87.31	1985.82	339.56	284.05	320.91
CSF CXCL13 (ng.L^-1^)	42	10.70	265.50	37.43	10.70	61.16
CIS						
Age (year)	14	16.00	44.00	28.57	27.00	8.86
CSF CHI3L1 (μg.L^-1^)	14	38.50	269.00	83.92	65.70	58.29
CSF NFL (ng.L^-1^)	14	139.00	1204.00	531.14	479.50	311.42
CSF pNFH (ng.L^-1^)	10	104.18	475.28	188.35	159.37	105.92
CSF CXCL13 (ng.L^-1^)	14	10.70	116.10	32.56	23.55	30.26
OIND						
Age (year)	11	21.00	85.00	56.00	56.00	19.11
CSF CHI3L1 (μg.L^-1^)	11	75.30	503.00	216.03	187.00	127.97
CSF NFL (ng.L^-1^)	7	161.00	10073.00	2055.43	690.00	3553.81
CSF pNFH (ng.L^-1^)	9	109.96	22400.00	3686.63	682.55	7161.84
CSF CXCL13 (ng.L^-1^)	7	< 10.70	80040.00	11464.36	24.10	30239.04
IDPNS						
Age (year)	4	37.00	73.00	53.50	52.00	15.35
CSF CHI3L1 (μg.L^-1^)	4	89.70	230.00	139.38	118.90	64.77
CSF NFL (ng.L^-1^)	4	354.00	27149.00	7430.75	1110.00	13151.77
CSF pNFH (ng.L^-1^)	4	226.88	23100.00	6121.19	578.93	11321.79
CSF CXCL13 (ng.L^-1^)	1	10.70	10.70	10.70	10.70	0.00
NIND						
Age (year)	46	12.00	79.00	54.07	55.00	16.08
CSF CHI3L1 (μg.L^-1^)	46	61.10	386.00	163.37	140.00	83.56
CSF NFL (ng.L^-1^)	31	210.00	60600.00	3997.87	744.00	10994.63
CSF pNFH (ng.L^-1^)	43	141.73	15500.00	1573.79	501.34	2892.56
CSF CXCL13 (ng.L^-1^)	24	10.70	96.20	17.01	10.70	21.57
Control						
Age (year)	15	18.00	67.000	41.200	39.000	15.8439
CSF CHI3L1 (μg.L^-1^)	15	32.30	188.00	85.84	76.90	42.24
CSF NFL (ng.L^-1^)	7	108.00	644.00	324.14	303.00	182.20
CSF pNFH (ng.L^-1^)	14	85.85	526.05	235.18	241.16	123.74
CSF CXCL13 (ng.L^-1^)	5	< 10.70	< 10.70	< 10.70	< 10.70	0.00

MS, multiple sclerosis; CIS, clinically isolated syndrome; OIND, other central nervous system inflammatory diseases; IDPNS, inflammatory diseases of the peripheral nervous system; NIND, non-inflammatory diseases of the central nervous system; n, number of patients; Min, minimal concentration; Max, maximal concentration; SD, standard deviation

**Fig 2:**
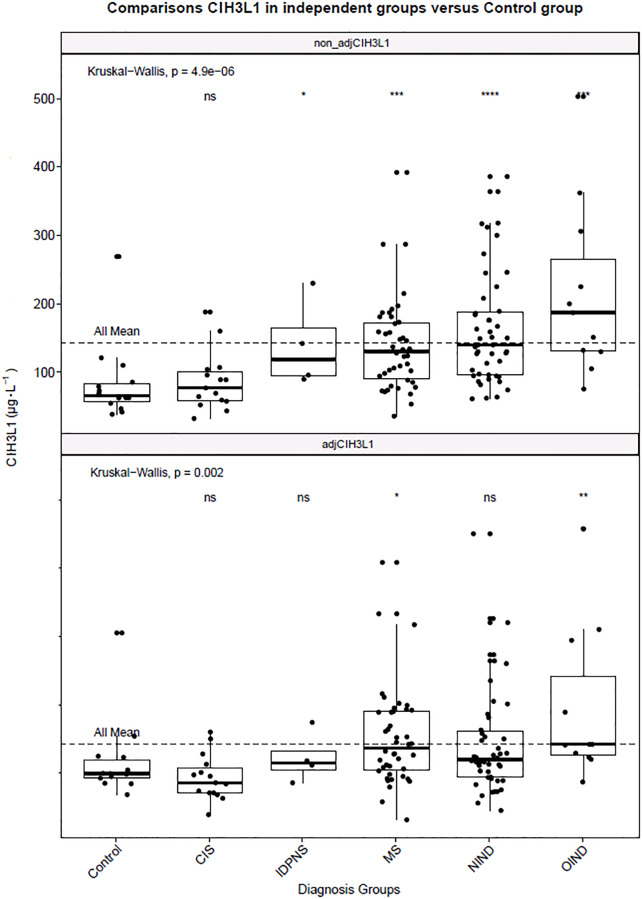
Box-plot concentration of CSF CHI3L1 individual diagnosis groups against base-mean CHI3L1 and results of pairwise comparisons between individual diagnosis groups against “control group”. Upper graph: non-adjusted levels of CHI3L1 Statistically significant differences were found in the IDPNS, MS, NIND and OIND groups. Lower graph: Age adjusted levels of CHI3L1. Statistically significant differences were found only in the MS and OIND group.

A statistically significant difference was found by the nonparametric Kruskal-Wallis test between CHI3L1 and diagnosis groups (p < 0.0001). After age-adjusted analysis according to regression dependence CHI3LI = 51.874 + 1.997 * Age, OIND diagnosis group had higher CHI3L1 levels than other diagnosis groups, (p = 0.002), Fig 2. Comparison of the individual groups against the Control was performed by the Wilcoxon test.

**Fig 3:**
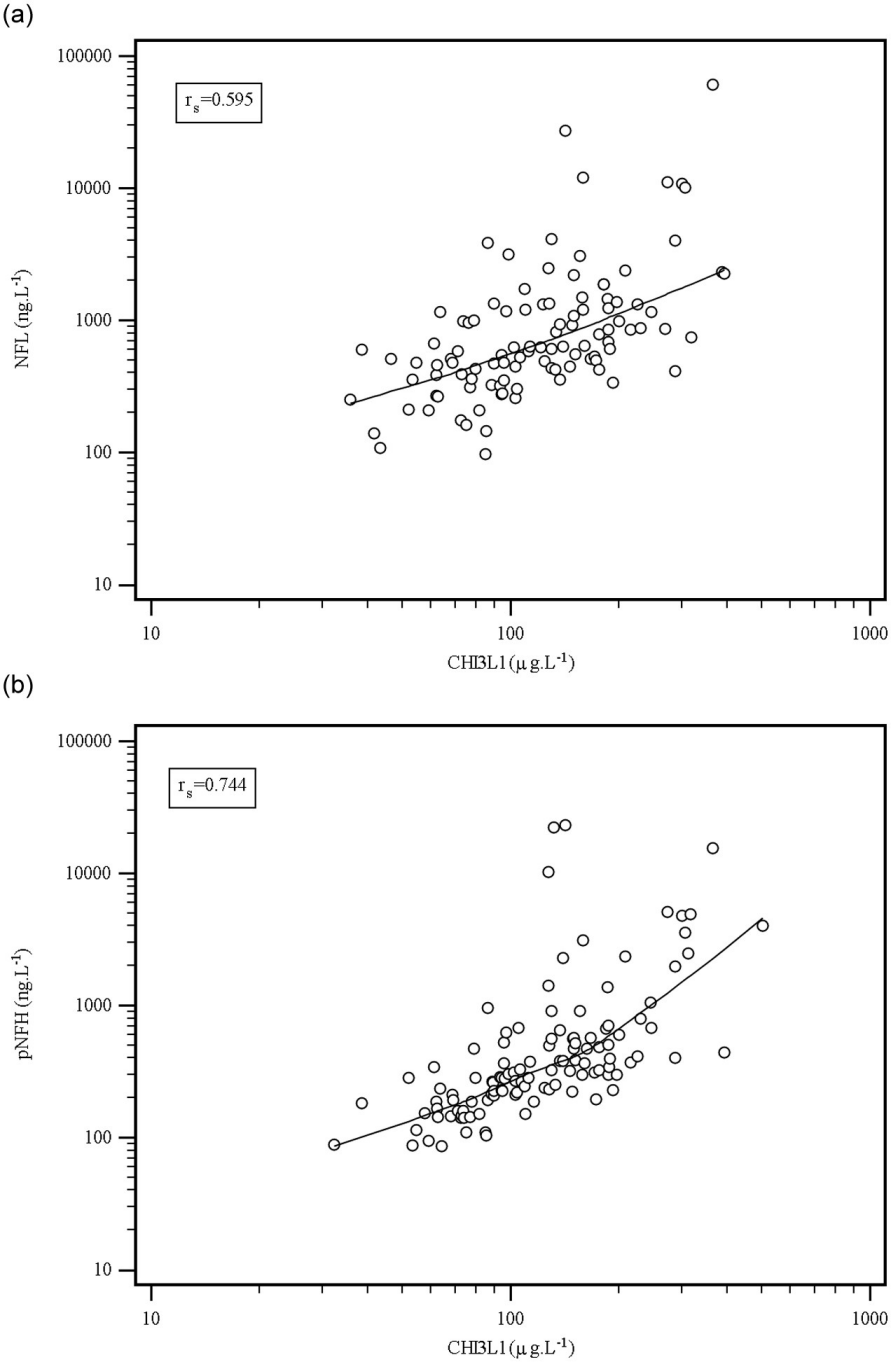
Passing-Bablok regression analysis of CHI3L1 and NFL, respectively pNFH concentrations in all groups. r_s_ = Spearman correlation coefficient. The correlation between CHI3L1 and NFL and pNFH in cerebrospinal fluid, P < 0.0001.

The regression relationship between studied parameters was evaluated using Passing–Bablok regression (Fig 3). There was a statistically significant correlation between CHI3L1 and NFL concentrations (r_s_ = 0.595; n = 105; P < 0.0001) and between CHI3L1 and pNFH concentrations (r_s_ = 0.744; n = 115; P < 0.0001).

At the same time, we evaluated the correlation between the CHI3L1 concentrations and other studied parameters in individual diagnostic groups. There was a statistically significant relationship between CHI3L1 and NFL in the MS (r_s_ = 0.460; P = 0.002), NIND (r_s_ = 0.503; P = 0.003), and OIND (r_s_ = 0.964; P < 0.001) diagnosis groups, and between CHI3L1 and pNFH in the MS (r_s_ = 0.691; P < 0.001) and NIND diagnosis groups (r_s_ = 0.691; P < 0.001) (Table 4). We performed a categorical comparison of the selected variables expressed in positivity or negativity of test with individual diagnosis using Fisher’s exact test. Positive values of the quantitative tests were as follows: CHI3L1 > 194.7 μg.L^-1^; NF-L > 900 ng.L^-1^ [18]; CSF pNFH > 610 ng.L^-1^ [19]; CXCL13 > 20 ng.L^-1^ [20], in the case of qualitative tests two extra oligoclonal bands (OCB) in CSF compared to serum (OCB ≥ 2) were the interpretation criteria for intrathecal oligoclonal IgG, IgM, and FLC synthesis. Statistically significant differences were found in pNFH, oIgG, oIgM, oFLC kappa, and oFLC lambda (P < 0.0001) (Table 5).

**Table 4:** Correlations between selected biochemical markers in CSF and the indicated diagnoses.

Parameters		Diagnosis					
		MS	CIS	NIND	OIND	Control	All
CHI3L1 vs. NFL	r_s_	0.460	0.422	0.503	0.964	0.857	0.595
	P	0.002	0.131	0.003	<0.001	0.0137	<0.0001
	n	42	14	31	7	7	105
CHI3L1 vs. pNFH	r_s_	0.691	-0.091	0.691	0.550	0.662	0.744
	P	<0.001	0.802	<0.001	0.125	0.010	<0.0001
	n	35	10	43	9	14	115
CHI3L1 vs. CXCL13	r_s_	0.302	0.421	0.391	0.371		0.205
	P	0.052	0.133	0.059	0.4131	n.a.	0.0482
	n	42	14	24	7		93

MS, multiple sclerosis; CIS, clinically isolated syndrome; OIND, other central nervous system inflammatory diseases, NIND, non-inflammatory diseases of the central nervous system, IDPNS, inflammatory diseases of the peripheral nervous system; r_s_, Spearman’s correlation coefficient; n.a., not applicable

**Table 5:** Categorical comparison of the selected variables expressed in positivity or negativity of test with individual diagnosis using Fisher’s exact test, because we compared quantitative methods (CHI3L1, NFL, pNFH, CXCL13) with qualitative methods (oligoclonal bands IgG, IgM, FLC kappa and FLC lambda).

Methods (n)	Fisher exact test P	Sample distribution	Diagnosis				
			CIS	MS	IDPNS	NIND	OIND
CHI3L1 (117)	0.0770	Neg	13	37	3	35	6
		Pos	1	5	1	11	5
CXCL13 (104)	0.0004	Neg	8	32	4	28	4
		Pos	9	11	0	2	6
NFL (106)	0.3775	Neg	13	25	2	19	4
		Pos	3	18	2	16	4
pNFH (104)	<0.0001	Neg	11	33	2	26	4
		Pos	0	2	2	18	6
oIgG (106)	<0.0001	Neg	5	5	8	18	9
		Pos	11	38	0	6	6
oIgM (55)	0.0001	Neg	3	7	1	17	3
		Pos	4	16	0	1	3
oFLC Kappa (132)	<0.0001	Neg	2	6	3	46	6
		Pos	13	37	1	10	8
oFLC Lambda (132)	<0.0001	Neg	7	15	3	53	9
		Pos	8	28	1	3	5

The Cohen´s kappa statistic was used to compare the assays based on clinical interpretation (positive and negative results) because the methods had different reference intervals (Table 6). The higher the kappa value, the greater the agreement between the methods. The highest kappa coefficient, i.e. moderate conformity between the studied biomarkers, was demonstrated between the concentrations of CHI3L1 and pNFH (< = 0.436).

**Table 6:** Correlation of selected parameters based on positivity and negativity of results, Cohen´s kappa statistics. Positive values were as follows: CHI3L1 > 194.7 μg.L^-1^; NF-L > 900 ng.L^-1^ [18]; CSF pNFH > 610 ng.L^-1^ [19]; CXCL13 > 20 ng.L^-1^ [20].

	CHI3L1 vs. NFL	CHI3L1 vs. pNFH	CHI3L1 vs CXCL13
Weighted Kappa (n)	0.243 (105)	0.436 (115)	0.287 (93)
95% CI	0.058–0.428	0.242–0.630	0.067–0.507
Standard error	0.0942	0.0988	0.112

95% CI, 95% confidence interval

The Mann-Whitney U test was chosen to compare parameters (CHI3L1, pNFH, NFL and CXCL13) with and without the presence of oligoclonal bands (oIgG, oIgM, oFLC; positive results indicate more than 2 bands in cerebrospinal fluid absent from serum). Statistically significant differences were not found with the presence of oligoclonal bands for CHI3L1, but were found with oIgG for pNFH and CXCL13 (P = 0.0061; resp. P <0.0001), with oIgM for CXCL13 (P = 0.0279), with oFLC lambda for NFL and CXCL13 (P = 0.0348; resp. P <0.0001), oFLC kappa and CXCL13 (P <0.0001).

## Discussion

In this study we tested CHI3L1 as a marker of multiple sclerosis. The CHI3L1 ELISA diagnostics kit is sensitive enough to measure CHI3L1 in cerebrospinal fluid. We also estimated the normal CSF CHI3L1 values that were show to be independent of gender but associated with age. The CHI3L1 concentration increased with increasing age. This is consistent with published data showing increased levels of CSF CHI3L1 in patients with CNS inflammation compared with healthy individuals, and an increase with increasing age, consistent with the hypothesis that lower-grade inflammatory processes are induced in the aging brain [9].

The dependence of CH3L1 concentration in different diagnostic groups was studied. An elevated level of CSF CHI3L1 was found in patients with MS, but it was much higher in patients with other inflammatory neurological diseases. Modvig et al. [21] reported similar results. They suggested that increased levels of CSF CHI3L1 are associated with tissue damage related to inflammation and might predict residual disabilities and possibly cumulative damage over time.

At the same time, a number of studies dealt with the study of CHI3L1 in relation to CNS tissue damage. Burman et. [8] found a fundamental difference in the origin of tissue damage in T1 lesions relative to normal appearing white matter, consistent with the dual paradigm that inflammation and degeneration are important for the development of tissue damage and disability in different form of MS. Baldacci et al. [22] conclude that CHI3L1 is a pathophysiological biomarker of neurodegeneration and its concentration correlate with parameters of neuronal injury, large axonal damage and synaptic disruption in different neurodegenerative disease.

Concurrently, CSF CHI3L1 concentration correlates significantly with CSF NFL and even more with CSF pNFH concentrations, making pNFH kit more convenient for routine analysis. The correlation between CHI3L1 and NFs varies depending on the diagnosis.

We also assessed the correlation between CHI3L1 and CXCL13. CXCL13 is a potent B cell chemoattractant. In cerebrospinal fluid, it is expressed by monocytes and especially macrophages in perivascular inflammatory lesions and scattered parenchymal cells [23]. Khademi et al. stress that this chemokine could be a very important marker of inflammatory activity in patients with multiple sclerosis [24]. The authors demonstrated a statistically significant correlation of elevated CXCL13 levels with the conversion of CIS to clinically definite MS and the rate of relapses one year after diagnostic lumbar puncture. They also found a significant relationship between high levels of CXCL13 and the presence of oligoclonal IgG bands in cerebrospinal fluid. This finding is confirmed by our observation of statistically significant differences in CXCL13 concentrations between oIgG, oFLC kappa and oFLC lambda negative versus positive patients.

The present study also provides the first results that compare CHI3L1, NFL, pNFH, and CXCL13 with IgG, IgM, and FLC kappa and FLC lambda oligoclonal bands. Our reason for conducting the comparison is the fact that intrathecal synthesis of IgG as well as of free light chains (FLC) is detectable in the majority of patients with multiple sclerosis and less frequently in other, mostly inflammatory, nervous system diseases. In addition, a positive finding of intrathecal FLC synthesis can be a marker of disease progression [25]. The detection of oIgM may help us to better understand the nature of the intrathecal antibody response in inflammatory neurological diseases and may be important in their differential diagnoses [26–27].

Since CSF CHI3L1 concentrations correlated much more closely with CSF pNFH, an established biomarker of disease progression than with inflammatory biomarker CSF CXCL13 and oFLC, we hypothesize that CSF CHI3L1 levels might reflect the extent of tissue damage rather than the degree of inflammatory activity. Lowest CSF CHI3L1 level found in the CIS group might further support this hypothesis, although patients with CIS (i.e., not fulfilling the diagnostic criteria of MS after the first episode consistent with demyelination) are probably at lower risk of progression to CDMS than before the implementation of 2017 revision of MS diagnostic criteria.

### Limits of the study

Our study has several weaknesses. Although three CSF and serum aliquots were frozen for each patient in the study, there was not always enough material, especially cerebrospinal fluid, for all tests. At the same time, the patient population was obtained during a 3-year study (n = 356), but in some patients no definitive diagnosis has been established. Therefore, these patients were not included in the final evaluation, which also affected the unequal representation of patients in the individual diagnostic groups. Finally, detailed clinical characteristics of patients within individual diagnostic groups were not taken into account in the analysis.

## Conclusions

In this study, we tested a diagnostic kit for the determination of CHI3L1 concentrations in biological fluids. The CHI3L1 ELISA assay has adequate sensitivity and is suitable for CSF analysis. The data showed good correlation and moderate conformity between CHI3L1 and pNFH concentrations. When assessing the relationship of CHI3L1 concentrations and diagnosis, correlations were found between the concentrations of CHI3L1 and NFL in the MS, NIND, and OIND groups, and between the concentrations of CHI3L1 and pNFH in the MS and NIND groups. The results support the hypothesis that CSF CHI3L1 could be another promising prognostic, albeit probably etiologically nonspecific, biomarker of MS.

## Supporting information

S1 Table Descriptive characteristics of the individual studied groups. (DOCX)

S1 Data (XLSX)

S2 Data (XLSX)
