# Supplementary material for: Determination of chitinase 3-like 1 in cerebrospinal fluid in multiple sclerosis and other neurological diseases
Source: PLoS One. 2020 May 21;15(5):e0233519. doi: 10.1371/journal.pone.0233519 (PMC7241789; doi:10.1371/journal.pone.0233519)
Supplement: S1 Table — (DOCX) [file pone.0233519.s001.docx]

**S1 Table. Summary descriptive characteristics of the studied group.**

| Variable | n | Min | Max | Mean | Median | SD |
| --- | --- | --- | --- | --- | --- | --- |
| Age (year) | 132 | 12.0 | 85.0 | 45.2 | 43.0 | 16.9 |
| CSF CHI3L1 (μg.L^-1^) | 132 | 32.3 | 503.0 | 142.2 | 125.5 | 84.3 |
| CSF NFL (ng.L^-1^) | 105 | 97.0 | 60600 | 2107.5 | 623.0 | 6638.6 |
| CSF pNFH (ng.L^-1^) | 115 | 85.8 | 23100 | 1238.2 | 311.8 | 3450.9 |
| CSF CXCL13 (ng.L^-1^) | 93 | 10.7 | 80040 | 889.8 | 10.7 | 8296.8 |

n, number of patients; Min, minimal concentration; Max, maximal concentration; SD, standard deviation
